# Supplementary material for: Local government debt and corporate tax burden: A perspective based on the trade-off of government tax collection and management
Source: PLoS One. 2023 Jul 12;18(7):e0287763. doi: 10.1371/journal.pone.0287763 (PMC10337903; doi:10.1371/journal.pone.0287763)
Supplement: Attachment — Submitted filename: review_R1.pdf [file pone.0287763.s002.pdf]

Submission Id: PONE-D-22-28623
Title: Local government debt and corporate tax burden: a perspective based on the trade-off of government tax collection and management

Dear Editors and Reviewers,
Thank you very much for your guidance and review of the article, and put forward very valuable review suggestions. These opinions have an important guiding role in the research design and theoretical analysis of the article, and greatly improve the article.
According to the suggestions of the reviewers, we have revised and replied each point which is being raised, and the revised part are marked in the file labeled 'Revised Manuscript with Track Changes'. The specific modification instructions are as follows:

For Reviewer #1: Review of „Local government debt and corporate tax burden"
The paper looks at the relationship between the development of public debt on regional level in China and the efforts to collect more taxes from corporations. The hypothesis tested is whether the municipalities have incentives to increase their collection efforts as a result in increases in public debt to reduce the fiscal pressure. The authors find, using a panel regression with fixed effects and an instrumental variables specification, that higher levels of public debt correlate positively with the tax burden of companies.
While the topic is interesting and relevant, the methodology of investigation is weak and does not support the conclusions. Moreover, the results are very context specific, which raises doubts regarding the external validity and thus interest for a general-interest journal such as Plos One. I do not recommend publication of the paper.

Major suggestions:
• Panel estimation: while the number of observations looks impressive, as the authors treat year-companies as a unit of observation, closer look reveals that the actual variation is much lower. Firstly, only five years are considered, which limits the time variation of the main explanatory variable. Secondly, and more importantly, local public debt is measures at the regional (local?) level (in fact, nowhere in the paper it is specified what the actual unit of observation of the debt variable is!). Implicitly, it seems that this is provincial data (e.g., referring to other provinces in the construction of the instrument), which means that the spatial variation is at 23 provinces or 34 regions. This does not allow for serious panel estimation. Moreover, this estimation requires an inclusion of regional fixed effects – which are not included. Why not?

Thanks to the reviewer for the suggestions. Limited by practical factors and data conditions, the research samples in this study are indeed not in a completely ideal research scenario. However, based on the reference and comparison of the existing literature, we believe that the empirical data in this study are based on the real and reliable realistic situation, and the research methods of the existing authoritative literature are used for reference as much as possible, which can provide empirical evidence for the research topic of this paper. The details are as follows:
(1)As China is in the stage of emerging and transition, the relevant systems of local government bond issuance mode are also in the process of continuous adjustment and improvement. Before 2015, China's central government did not strictly control the bond issuance mode of local governments, and local governments had a variety of hidden financing channels, resulting in multiple statistical caliber of local government debt data, and the data were not open and

transparent to the public. Since 2015, China's new Securities Law has been implemented, which strictly requires local governments to borrow debt by the "self-issue and self-repayment" model. And the relevant data are disclosed on China Government Debt Center (http://www.celma.org.cn/), which is more authoritative and reliable than the estimated and manually collected data in previous literature. Therefore, we have to define the research interval from 2015 to 2019. Although the research interval is short, a total of 12,325 company-year observations are provided by 31 provinces and regions in the five years, which can provide sufficient empirical evidence for the research of this paper.

(2)This paper explains the scope of the sample in the sample selection section of the research design. Specifically, the samples come from the debt balance information of all provinces and cities in China since 2015, including 23 provinces, 4 municipalities directly under the Central Government and 5 autonomous regions. Combining the data of local government debt at the macro level with the data of enterprises at the micro level, this paper examines the microeconomic consequences of local government debt by referring to many authoritative literature, such as Croce et al. (2019), Liang et al. (2017) and Rao et al. (2022), which can provide empirical evidence for the study.

The main reasons for not including regional fixed effects are as follows:
In China, the statutory corporate tax rate and tax categories are uniformly set by the central government, and there are no significant differences between different regions of the country. Changes in the actual tax burden level will be around the statutory tax rate level, and the degree of fluctuation is small, so it is difficult to observe changes in the tax burden level of enterprises in the same region in each year. In addition, this paper intended to use the differences between different regions to observe the tax trade-off of different local governments. The setting of the model can help us to start from the overall perspective and observe that when the statutory tax rate is basically the same, the regions with larger local government debt have higher actual tax burden. Based on the above considerations, this paper does not include regional fixed effects. However, we controlled macro-level factors as much as possible, such as regional population, investment status and regional economic status. Moreover, the standard errors are clustered to the provincial and municipal levels, which can control the related interference factors to a certain extent.

[1] Liang Y, Shi K, Wang L, Xu J. Local Government Debt and Firm Leverage: Evidence from China. Asian Econ Policy Rev. 2017; 12(2):210-232. https://10.1111/aepr.12176.
[2] Croce MM, Nguyen TT, Raymond S, Schmid L. Government debt and the returns to innovation. J Financ Econ. 2019; 132(3):205-225. https://10.1016/j.jfineco.2018.11.010.
[3] Rao PG, Tang S, Li XX. The Crowding-Out Effect of Local Government Debt:Evidence from Corporate Leverage Manipulation. China Industrial Economics. 2022;(01):151-169. https://10.19581/j.cnki.ciejournal.2022.01.009.

Thank you for clarifying the limited sample size. Time restriction now makes more sense to me. While I am still not entirely convinced by the fact that there is very little variation, it is good to see that the coefficients remain significant when clustering at the provincial level. However, as authors mention, there is little to no variation of the main independent variable over time

(which is why they do not include fixed effects), the empirical exercise is essentially a cross-unit regression on 32 units of observations. Such a set-up could be very sensitive to outliers. You should provide some tests, e.g., residuals vs leverage plots or (better) as an additional robustness "leave-one-out" regressions.

• Related to the previous point is the question of endogeneity. An obvious candidate for a confounder is the overall economic activity in a region, which affects both the denominator of the debt ratio and (indirectly) the denominator of the tax burden, e.g., through operating income or pre-tax profit. This means, that any changes to the economic situation of each province, will likely affect both sides of the regression equation. Authors use in one specification a Hausman-type spatial instrument, which replaces own debt levels, with an average of debt levels of other provinces. This instrument does not resolve the endogeneity issue, as any idiosyncratic change to the economic situation of all regions still affects both the explanatory and the outcome variables. Besides, no test statistics of the instruments, such as first-stage regressions, or the overidentification tests, F-Statistics and like to assess the quality of the instrument are provided.

Thanks to the reviewer for the suggestions.
Drawing on the existing research (Demirci et al., 2019; Chongi et al., 2013), we adjusted the instrumental variables for the articles:
we use the mean value of education expenditure of other provinces and cities in that year as the instrumental variable (Espend). On the one hand, the evaluation system of local officials in China includes the inspection of people's livelihood matters, so there is competition among local governments in the scale of fiscal expenditure on people's livelihood (Lu et al., 2022). That is, when other regions increase fiscal expenditure to improve people's livelihood performance, local governments will increase fiscal expenditure accordingly for regional competition. Moreover, there is a certain correlation between government revenue and expenditure and local government debt scale, which meets the correlation requirements of instrumental variables. On the other hand, education expenditure is the basic expenditure of local finance on people's livelihood, which belongs to the local rigid expenditure and has a weak correlation with the short-term economic fluctuations that affect the actual tax burden of enterprises. This means Espend is relatively exogenous. At the same time, the pressure of fiscal expenditure in other regions will not be directly transferred to enterprises within the jurisdiction. All the above characteristics enable Espend to meet the exogeneity requirements of instrumental variables.
Table 6 reports the regression results of the instrumental variables in Column (1)-(4). And the instrumental variable (Espend) passed the underidentification test and the weak identification test.

Table 6. Testing for endogeneity.
(1) (2) (3) (4)
first second first second
Variables GovDebt1 Taxburden1 GovDebt1 Taxburden2
Espend 0.002*** 0.002***
(7.47) (7.67)

GovDebt1 0.0766*** 0.0625**
(3.98) (2.98)
Namerate 0.029 0.559*** 0.003 0.117***
(0.67) (13.68) (0.10) (6.14)
Size 0.000 -0.002 -0.001 0.002**
(-0.09) (-1.51) (-0.68) (2.72)
Roa -0.053 -0.608*** -0.034 0.268***
(-1.41) (-11.63) (-0.97) (11.97)
BM -0.004 0.014 0.007 -0.002
(-0.60) (1.91) (0.97) (-0.49)
Lev 0.010 0.011 0.010 -0.051***
(1.13) (1.32) (1.17) (-11.61)
Tangible 0.006 0.002 0.008 -0.008
(0.54) (0.18) (0.68) (-1.38)
Intangible -0.023 0.056 -0.020 0.057**
(-0.73) (1.71) (-0.71) (3.08)
Invent -0.021 0.034** -0.022 -0.011
(-1.20) (2.71) (-1.34) (-1.20)
Loss 0.004 0.036*** 0.005 -0.004*
(1.49) (4.43) (1.72) (-2.11)
Top10 -0.014 -0.003 -0.009 0.010*
(-1.75) (-0.39) (-0.91) (2.25)
Indep 0.025 0.049** 0.016 0.006
(0.79) (2.74) (0.68) (0.47)
Density -0.273*** -0.002 0.000*** 0.000
(-3.66) (-0.30) (-3.74) (-1.88)
Inv 0.208 -0.058 0.132 -0.130***
(0.58) (-0.83) (0.38) (-3.61)
GDPr -0.345** 0.014 -0.354** 0.018
(-3.05) (0.67) (-3.12) (1.35)
Constant -1.531*** 0.041 -1.495*** -0.037*
(-6.27) (1.13) (-6.69) (-1.99)
Year/Industry YES YES YES YES
Observations 12,325 12,325 9,390 9,390
R2 0.263 0.302
Adj-R2 0.536 0.564
Underidentification test:
Kleibergen-Paap rk LM statistic 5.055** 5.275**
Weak identification test:
Kleibergen-Paap Wald rk F statistic 55.84*** 58.84***
Overidentification test of all instruments:
Hansen J statistic equation exactly identified equation exactly identified
Notes: *, ** and *** indicate statistical significance at the 10, 5, and 1% levels, respectively. The values in parentheses in columns (1) and (3) are t-statistics, and the values in parentheses in columns (2) and (4) are z-statistics. The standard errors are clustered at the provincial and municipal levels (31 groups) by using the wild cluster bootstrap method.

[1] Demirci I, Huang J, Sialm C. Government debt and corporate leverage: International evidence. J Financ Econ. 2019; 133(2):337-356. https://10.1016/j.jfineco.2019.03.009.
[2] Chong TT, Lu L, Ongena S. Does banking competition alleviate or worsen credit constraints faced by small- and medium-sized enterprises? Evidence from China. J Bank Financ. 2013; 37(9):3412-3424. https://10.1016/j.jbankfin.2013.05.006.
[3] Lu J, Cai S, Zang T. Strategic Competition of County-level Governments under Promotion Game:Evidence from Zhejiang Province. Finance and Trade Research. 2022; 33(05):1-14. https://10.19337/j.cnki.34-1093/f.2022.05.001.

I like the idea of the education expenditure instrument and it is good that you now provided some diagnostic tests. I do not particularly like the wording "testing for endogeneity" – you cannot test for endogeneity – but the contents of the table are solid.

• The overall empirical exercise is very context specific. The authors mention, that the channel of transmission for the higher tax burden is essentially only the tax collection effort, as the taxation rates and bases as such as regulated nation-wide. This raises the question of external validity – what can we learn from the paper on the similar cases all over the world?

Thanks to the reviewer for the suggestions.

Indeed, this paper takes the realistic situation in China as the specific research background, but the phenomena involved in this paper, such as government intervention, taxation trade-off, and regional competition, exist widely in all countries in the world (including developed and developing countries). With the help of the realistic situation of China, a representative developing country, this article enriches and expands the literature in the above fields, which has important theoretical and practical significance. The details are as follows:

Taking the actual tax burden level of micro enterprises as the research object, this paper expands the research on the economic consequences of local governments' expanding bond issuance in developing countries. At the same time, through mechanism test, heterogeneity analysis and other further tests, this paper reveals the role of local governments' intervention behavior in this process, analyzes the trade-off of local governments' taxation under different institutional environments, and enriches the literature in tax collection, regional competition and other related fields. It provides policy enlightenment for developing countries with large differences in regional development levels to improve public debt management system, create a fair tax environment, and promote high-quality economic growth, which has important theoretical and practical significance.

In addition, we have added a description of the above research contributions at the end of the article.

It is now more clear how the research adds to the literature.

• Hypotheses 2 and 3 have left me a bit puzzled. The authors suggest that the impact of debt on tax burden should be *more significant* in non-state-owned enterprises. I would think that there should *only* be an effect in those companies, unless there is something about China, which makes is "normal" that state-owned entreprises do not fulfill their tax obligations. If this is indeed the case, it needs to be explained in more detail.

Thanks to the reviewer for the suggestions.
As mentioned in the article, there is a natural blood relationship between state-owned enterprises and the government in China. Many studies have shown that (Chen et al., 2016; Liu and Hou, 2017; Yan et al., 2021), on the one hand, state-owned enterprises undertake a large number of political tasks such as public services, national defense construction, people's livelihood improvement, and stable employment. Therefore, the government will provide more policy compensation to them, including providing more tax incentives and a more relaxed tax collection environment. On the other hand, Chinese state-owned enterprises have strong tax lobbying capabilities. In order to ensure the financial flexibility and daily operation of enterprises, they will seek support from local governments to reduce the tax burden when necessary. And local governments are willing to provide a more lenient tax environment to state-owned enterprises in order to protect them and ensure their continued operation. Therefore, there is also room for adjustment of the tax burden of state-owned enterprises in China.

[1] Chen D, Kong MQ, Wang HJ. Economic cycle and tax avoidance of state-owned enterprises. Management World. 2016;(05):46-63. https://10.19744/j.cnki.11-1235/f.2016.05.006.
[2] Liu SY, Hou DC. The Measurement of Tax Bargaining Power of Listed Companies and Government——An Empirical Study Based on Bilateral Stochastic Frontier Model. Taxation and Economy. 2017;(04):87-95.
[3] Yan JM, Liu JJ, Yan H. Tax Burden and Tax Preferences from the Perspective of Tax Neutrality in China. Taxation and Economy. 2021;(3):22-31.

This is now more clear.

Minor:

Table 6 is beyond the margins and the righthand side is, thus, missing.
